# Current investigations for liver fibrosis treatment: between repurposing the FDA-approved drugs and the other emerging approaches

**DOI:** 10.3389/jpps.2023.11808

**Published:** 2023-11-07

**Authors:** Omima S. Mohammed, Hany G. Attia, Bassim M. S. A. Mohamed, Marawan A. Elbaset, Hany M. Fayed

**Affiliations:** ^1^ Department of Microbiology, College of Medicine, Najran University, Najran, Saudi Arabia; ^2^ Department of Pharmacognosy, College of Pharmacy, Najran University, Najran, Saudi Arabia; ^3^ Department of Pharmacology, Medical Research and Clinical Studies Institute, National Research Centre, Cairo, Egypt

**Keywords:** liver fibrosis, anti-fibrotic agents, HSCs, therapeutic targets, pharmacotherapy

## Abstract

Long-term liver injuries lead to hepatic fibrosis, often progressing into cirrhosis, liver failure, portal hypertension, and hepatocellular carcinoma. There is currently no effective therapy available for liver fibrosis. Thus, continuous investigations for anti-fibrotic therapy are ongoing. The main theme of anti-fibrotic investigation during recent years is the rationale-based selection of treatment molecules according to the current understanding of the pathology of the disease. The research efforts are mainly toward repurposing current FDA-approved drugs targeting etiological molecular factors involved in developing liver fibrosis. In parallel, investigations also focus on experimental small molecules with evidence to hinder or reverse the fibrosis. Natural compounds, immunological, and genetic approaches have shown significant encouraging effects. This review summarizes the efficacy and safety of current under-investigation antifibrosis medications targeting various molecular targets, as well as the properties of antifibrosis medications, mainly in phase II and III clinical trials.

## Introduction

Liver fibrosis results from a continuous scarring response caused by persistent tissue damage [1]. The main damaging etiologies are chronic hepatitis B (HBV) or hepatitis C virus (HCV) infection, alcohol misuse, non-alcoholic fatty liver disease (NAFLD), non-alcoholic steatohepatitis (NASH), and other illnesses like Wilson’s disease, autoimmune hepatitis, biliary cholangitis and hemochromatosis [2].

Deathly complications of cirrhosis include liver cancer, hepatic encephalopathy, ascites, systemic infection, and functional liver failure [3]. Currently, there are no licensed treatments for advanced fibrosis. However, ongoing clinical research is promising and shows the possibility of reversal of liver fibrosis [4].

Hepatic disease complicated by fibrosis is a primary reason for liver transplantation [5]. Moreover, 2 million people yearly lose their lives due to liver disease, with cirrhosis being the eleventh largest cause of death globally [6].

The present article will review the mechanism of liver fibrosis and evidence-based pharmacological strategies attempting to treat this devastating liver pathology.

## Biological mechanism of liver injury

Chronic exposure to external pathological factors can harm hepatocytes, stimulate the infiltration of lymphocytes, activate inflammatory cells like macrophages, and ultimately activate hepatic stellate cells (HSCs), transforming them into myofibroblasts, consequently producing too much extracellular matrix (ECM) which causes liver fibrosis and scarring [7]. In normal livers, HSCs reside in the perisinusoidal region and remain dormant quiescent HSCs (qHSCs). Myofibroblasts (activated HSCs, aHSCs) are known for synthesizing ECM components like fibrillar collagen types I and III, as opposed to the laminar types IV and VI, mostly common in healthy liver tissue. This trans-differentiation occurs as a result of a complex activation. This complex activation occurs as a reaction to several signals that promote fibrosis, such as signals emanating from hepatocytes that have been harmed, growth factors produced by Kupffer cells, and changes in the ECM. Liver fibrosis is believed to be associated with the activation of HSC. The quantity of aHSCs diminishes following the completion of injury treatment, potentially impeding or stopping the advancement of liver fibrosis. It is hypothesized that treating liver fibrosis may be associated with a reduced interaction between HSCs and other injured hepatic cells [4, 8].

Furthermore, activated HSCs exhibit enhanced contractility, have high levels of “alpha-smooth muscle actin (α-SMA), and release cytokines such as transforming growth factor-beta 1 (TGF-β1), platelet-derived growth factor (PDGF) and connective tissue growth factor (CTGF) [9]. aHSCs are continuously stimulated by their own autocrine. Moreover, chemotactically moving to the region of the injured liver, chemokines secreted by aHSCs build up in the body’s inflammatory system, aggravating the injury caused by inflammation. Additionally, Damage-Associated Molecular Patterns (DAMPs) released by damaged hepatocytes activate “Kupffer cells” and a variety of immunological cells, which in turn activate HSCs and retain their existence by discharging pro-(inflammatory and fibrogenic) besides, the activation of the TGF-β1/Smad signal pathway, mitogen-activated protein kinase (MAPK) signal pathway and other signal pathways” [10].

In addition, CC chemokines (chemokine (C-C motif) ligand 2 (CCL2)-(CCL5)), which attract leukocytes to damaged sites, are secreted by Kupffer cells. Further, monocytes secrete mediators including Apoptosis-signal-regulating kinase 1 (ASK1), Pan-caspase, Galectin-3 (Gal-3), and other chemicals, which further harm hepatocytes, aggravate inflammation, encourage HSC activation and fibrosis [11, 12]. Moreover, TGF-β1 promotes monocytes to develop into macrophages. Macrophages, for example, secretes interleukin 1 (IL-1) and interleukin 6 (IL-6), which contribute to the exacerbation of the inflammatory reaction and the ongoing activation and survival of HSCs [13]. The initiation of HSCs is influenced by the paracrine crosstalk among macrophages and Kupffer cells.

## Animal models to study liver fibrosis

An experimental study in rats is currently the gold standard experiment in liver fibrosis research to support a proposed disease-associated mechanism resembling clinical scenarios. Based on etiology, there are now multiple categories for *in vivo* models of liver fibrosis, including genetically modified, chemical, nutritional, surgical, and infection-based models.

Carbon tetrachloride (CCl_4_), ethanol, thioacetamide (TAA), dimethylnitrosamine (DMN), and diethylnitrosamine (DENA) are substances that are frequently used to induce liver fibrosis and cause hepatic diseases. Further, the progression of NAFLD to hepatic fibrosis in experimental animals can be rendered using a variety of specialized diets, including the methionine- and choline-deficient or high-fat diet. Moreover, common bile duct ligation (BDL) can cause periportal biliary fibrosis and cholestatic damage.

## Liver fibrosis treatment plans

The primary focus of current liver fibrosis treatment plans is the eradication of etiologies. Lifestyle modifications and bariatric surgery have been studied for hepatic metabolism problems [34], and antiviral medications for viral hepatitis [35, 36] have all produced clinical evidence for resolving hepatic fibrosis, indicating that scarring is reversible.

Indeed, it was demonstrated that repurposing FDA approved drugs or experimental molecules (Tables 1, 2) for targeting the hepatic cells interaction implicated in excessive collagen deposition can be accomplished through out the following strategies [37, 38].

**TABLE 1:** Examples of ongoing research regarding repurposing FDA-approved drugs and using experimental drugs in the treatment of liver fibrosis.

FDA-approved drug repurposing	Proposed target	References	Investigational stage
Aspirin	TLR4/NFκB- TGFβ	[14]	Preclinical
Liraglutide	GLP-1	[15]	Phase III
Losartan	Angiotensin II	[16]	Phase IV
Pentoxifylline	TNFα	[17]	
Pirfenidone	TGFβ	[18]	Phase II
Praziquantel	SMAD7	[19]	
Sorafenib	PDGFR (platelet-derived growth factor receptor)/HIF-1α	[20]	Phase III
Pioglitazone	CTGF(connective tissue growth factor)	[21]	Preclinical
Octreotide	Wnt/β-catenin	[22]	Preclinical
Statins	HMG CoA Reductase	[23]	Phase II
Sitagliptin	Dipeptidyl Peptidase-4	[24]	
Rosiglitazone	PPAR gamma	[25]	Phase II
Erythropiotein	ROS/cytokines	[26, 27]	Preclinical

**TABLE 2:** Examples of current studies investigating the utilization of FDA-unapproved medications and experimental drugs for managing liver fibrosis.

FDA-unapproved medications and experimental drugs	Proposed targets	References	Investigational stage
Cenicriviroc	CCR2/5	[28]	Phase III +ve
Resmetirom	THRβ	[29]	Phase III +ve
Emricasan	Pan-caspase	[30]	Phase II
Selonsertib	ASK1	[31]	Phase II
Hydronidone	FGFR1	[32]	Phase II +ve
Cilofexor	FXR	[33]	Phase II +ve

### Hepatic protection from hepatocyte death (apoptosis inhibition)

One of the main initiators of HSC activation and inflammatory reaction in all etiologies is hepatocyte cell death through apoptosis [39]. Hence, in animal models of hepatic fibrosis, suppression of hepatocyte apoptosis hinders HSC activation [40].

Preclinical [41] and clinical research [42] have used emricasan, the pan-caspase apoptosis inhibitor. Emricasan slowed the progression of fibrosis and liver damage in a NASH mouse model [43]. The BDL model of biliary fibrosis similarly showed increased survival and decreased portal hypertension with emricasan therapy [44]. In patients with advanced stages of hepatic cirrhosis and portal hypertension, emricasan reduces the model for end-stage liver disease “MELD” scores [45]. The effectiveness of emricasan in treating NASH patients is currently being studied “NCT03205345 ENCORE-LF; NCT02960204 ENCORE-PH, NCT02686762 ENCORE-NF” [46].

ASK-1 (apoptosis signal-regulating kinase 1) is repressed via the drug selonsertib. When there is oxidative stress, ASK-1 induces apoptosis and enhances the production of inflammatory cytokines [47]. In a rodent NASH animal model, selonsertib reduced liver fibrosis [48]. According to a rodent NASH model, selonsertib improved inflammation, steatosis, fibrosis, and metabolic markers linked to NAFLD. In the DMN-induced fibrosis model, selonsertib decreased the accumulation of collagen, expression of type I collagen, fibronectin, and α-SMA [31]. Following promising results from clinical trials involving hepatic steatosis patients, the effectiveness of selonsertib as an anti-fibrotic medication has been evaluated in two phase III clinical trials [49].

TNF-α causes acute liver failure and hepatocyte death [50]. Hepatocyte death is accompanied by the formation of apoptotic bodies, which are absorbed through Kupffer cells. This increases the synthesis of death ligands TNF-α, Fas ligand (FasL), and Tumor necrosis factor-related apoptosis-inducing ligand (TRAIL), causing more hepatocyte deaths [51].

In a prior trial, individuals with chronic hepatitis C were treated with pirfenidone (PFD), a drug approved for treating lung fibrosis, for 24 months; PFD reduced liver fibrosis and inflammation [52]. Further, according to new research, PFD reduced liver fibrosis in mice fed a high-fat diet and lacking the melanocortin 4 receptor in a mice-model of NASH. The Melanocortin 4 receptor (MC4R) has been implicated in developing and progressing liver diseases, notably NASH and hepatocellular carcinoma (HCC). PFD also inhibited TNF-α provoked liver cell programmed cell death with decreased caspase 8 and 3 activation, indicating that PFD employs suppression of hepatocyte death and anti-fibrotic effects in non-alcoholic steatohepatitis [53]. Recent findings from a clinical trial “NCT04099407” on the impact of PFD medicine on fibrosis and its safety for 1 year in cases with persistent liver disorders show that 35% of those receiving PFD experienced a substantial reduction in fibrosis [54].

### Oxidative stress reduction provides hepatic protection

Reactive oxygen species (ROS) and oxidative stress have significantly contributed to the onset of fibrogenesis by activating HSCs. Consequently, reducing these stressors and ROS reduces inflammation, which improves liver fibrogenesis. Antioxidants are emerging as possible anti-fibrotic treatments because they can reduce ROS production. As a result, several antioxidants are being examined in clinical studies with positive results, including “S-adenosyl-L-methionine (SAMe), silymarin, phosphatidylcholine, resveratrol, quercetin, N-acetylcysteine (NAC), s-allylcysteine (SAC), oroxylin A, methyl ferulic acid (MFA), vitamin E” and so on [55, 56]. In animal models and cell cultures, quercetin, daidzein, resveratrol, cyperus, curcumin, thymol, apigenin, rice bran oil, red yeast rice golden berry, and N-acetylcysteine (NAC) have effectively prevented liver injury and inhibited stellate cell activation [37, 57–66].

Reactive oxygen species (ROS) are frequently produced by NADPH oxidase (NOX) [67], and NOX1, 2, and 4 are crucial for HSC activation [68]. Setanaxib, previously named GKT137831, an inhibitor of NOX1, NOX4 inhibitor, and NADPH oxidase, decreases reactive oxygen species, nitric oxide, and fibrotic gene expression in CCL_4_ liver fibrosis in mice [69]. A recent phase II clinical trial introduced Setanaxib as an anti-cholestatic and anti-fibrotic agent for primary biliary cholangitis “NCT03226067” [70].

### Hepatic protection via gut microbiome restoration

Bacteria, protozoa, fungi, and viruses make up the gut microbiota in the human digestive tract. The gut microbiota is responsible for keeping the immune system in balance, preventing autoimmune, and preventing and eradicating pathogen invasion [71]. The pathophysiology of obesity and NAFLD/NASH is influenced mainly by the gut microbiota, particularly Firmicutes, and Bacteroidetes, with a relative decline in Firmicutes and an increase in Bacteroidetes [72]. Probiotics (*Lactobacillus reuteri*) and metronidazole medication, either alone or combined with metformin, may be an effective therapeutic strategy for treating NASH rat models by altering the gut microbiome [73]. Given the pathophysiological importance of gut dysbiosis to fibrogenesis, several studies have looked at the use of microorganisms (Probiotics), functional ingredients (Prebiotics), and fecal microbiota transplantation as anti-fibrotic therapies [72]. Prebiotics are indigestible food components, and probiotics are living microorganisms claimed to help or rebuild the intestinal microflora. Microorganisms functional ingredients have demonstrated protective benefits on NASH and liver toxicity in animal models of persistent hepatic damage, confirming the pathogenic importance of gut dysbiosis in chronic liver disorders [72]. A meta-analysis of VSL#3, the most extensively studied probiotic supplement in NASH/NAFLD patients, revealed possible anti-inflammatory and insulin-sensitizing benefits consistent with the preclinical evidence [74].

### Lipid-lowering agents for hepatic protection

Statins are inhibitors of 3-hydroxy-3-methyl-glutaryl-coenzyme A (HMG-CoA) reductase, which controls the rate-limiting step in hepatocyte cholesterol biosynthesis [75]. Statins have been demonstrated to have anti-inflammatory and anti-fibrotic activities in various animal models representing chronic liver disease [76]. According to two recent studies using retrospective cohorts, statins may help reduce fibrosis and steatosis and aid in avoiding disease progression in persons with NAFLD [77, 78]. But to verify their effectiveness, prospective trials are necessary. Steatosis and the NAFLD activity scores were decreased in participants who took atorvastatin [79]. According to emerging scientific and clinical data, statins may prevent fibrosis and delay the course of chronic liver disease “NCT03780673; NCT02968810; NCT04072601” [12].

### Prevention of HSC activation

When hepatic fibrosis develops and progresses, one of the most crucial stages is the activation of HSCs. Liver damage stimulates dormant HSCs to become active. Kupffer cells and other cells constantly activate HSCs via PDGF, TGF-β1, CTGF, and other mediators that stimulate HSC proliferation and extend HSC longevity through associated signaling pathways. In addition, HSCs’ autocrine function initiates its own activation [11]. The stimulation of HSCs is also influenced by several proteases, including dipeptidyl peptidase 4 (DPP4) and HMG-CoA reductase. Therefore, stopping HSC activation and proliferation is essential for lowering or reversing hepatic fibrosis. A recent animal study showed that linagliptin, a DPP4 family member, could decrease acute hepatic injury via modulation of C/EBP-β and CX3CL1/Fractalkine [80].

#### TGF-β1/Smad signal pathway

Stimulating the TGF-β1/Smad signal pathway is a critical step in advancing liver fibrosis. The type I receptor is attracted following serine residue phosphorylation and activated by TGF-β1 in HSCs after liver injury. Smad2/3 is a receptor-regulated protein that is re-phosphorylated by an active type I receptor and then dissociates from the receptor to combine with Smad4. This complex undergoes nuclear translocation and inhibits TGF-β1 via negative feedback by repressing the expression of Smad7, which controls the expression of genes associated with fibrosis, activation of HSC, stimulation of the overproduction and deposition of extracellular matrix, and aggravates fibrosis [81]. As a result, reducing liver fibrosis and stopping HSC activation and proliferation requires blocking the TGF-β1/Smad signal pathway.

Fluorofenidone [1-(3-fluorophenyl)-5-methyl-2-([1H])-pyridone] is a recent pyridone anti-fibrosis molecule that has shown a notable therapeutic effect on fibrosis. Such an effect was prominently demonstrated in the kidney, liver, and lungs [82]. Fluorofenidone reduced the TGF-β1-induced initiation of HSCs caused by and inhibited the TGFβ1-1/Smad and MAPK signaling, reducing hepatic damage or fibrosis triggered by pig serum in rodents [83]. Moreover, praziquantel, a schistosomicide with reasonable safety, markedly reduced collagen formation, up-regulated Smad7 expression in HSCs, inhibited the TGF-β1/Smad signal pathway, and hindered the stimulation of HSCs in mice with liver fibrosis caused by CCl_4_ [84].

#### Peroxisome proliferator-activated receptors (PPARs)

The superfamily of ligand-activated transcription factors known as PPARs tightly regulate energy homeostasis and metabolic activity. Peroxisome proliferator-activated receptor gamma (PPARγ) is predominantly regarded as a prospective target of liver fibrosis treatment among the three isoforms of PPARs (PPARα, PPARγ, and PPARα/δ)). The PPARγ expression reduces HSC activation, even though it is abundantly expressed in qHSCs [85].

Better steatosis and lobular inflammation were observed in a randomized clinical study of the insulin sensitizer PPAR receptor activator pioglitazone in individuals with non-cirrhotic NASH. Still, no meaningful effect on fibrosis was observed [86]. Treatment with pioglitazone for up to 2 years was related to fibrosis enhancement at any phase and remission of NASH, according to a following meta-analysis of 8 clinical trials for PPAR receptor activator treatment [87].

Elafibranor (GFT505) is a dual PPARα/δ agonist produced by Genfit in France [88]. Elafibranor improves the lipid profile, increases insulin sensitivity, and has anti-inflammatory and anti-fibrotic properties, among other things, which all work together to reduce the symptoms of NASH [89]. It has been shown in animal experiments that elafibranor administration helps to lower hepatic steatosis, inflammation, fibrosis, and the level of biomarkers for liver dysfunction, as well as in preventing the production of pro-inflammatory and pro-fibrosis genes [90]. Preventative and curative effects of elafibranor treatment for CCl_4_-induced hepatic fibrosis in rats [91]. Subgroup analysis from the phase III clinical study suggests that elafibranor may effectively treat those with severe NASH [92].

#### The farnesoid X receptor (FXR)

A nuclear receptor family member is crucial for controlling the metabolism of bile acids, lipids, and glucose. It was a desirable target because it regulates bile acid balance in treating cholestatic diseases [93]. The liver, small intestine, and HSCs express FXR at significant levels. Fascinatingly, overexpression of FXR prevented HSCs from producing collagen [94]. According to recent studies, FXR agonists may effectively treat liver illnesses other than cholestatic ones. In mouse NASH models, the FXR agonist WAY-362450, for instance, decreased liver fibrosis and inflammation [95]. FXR agonist also reduced hepatic fibrosis in several different hepatic fibrosis models (BDL, CCL_4_, and porcine models) by inducing small heterodimer partner (SHP) gene expression [96].

Obeticholic acid (OCA), a bile acid derivative, is a potent FXR activator that lowers liver fat and fibrosis in animal models of fatty liver disease [97]. In a phase 2 experiment, cases with diabetes mellitus type 2 and NAFLD received OCA at 25 or 50 mg once daily for 42 days. This therapy reduced liver fibrosis and inflammation markers while increasing insulin sensitivity “NCT00501592” [98].

#### Wnt/β-catenin signaling

According to studies, the initiation of HSCs and hepatic scarring are linked by the Wnt/β-catenin signal pathway. Wnt protein, along with frizzled receptor and lipoprotein receptor-related protein (LRP)-5/6, forms a complex that prevents the breakdown of β-catenin. In the presence of coactivators such as cyclic AMP response element-binding protein (CREB), β-catenin is activated, accumulates, and is then easily located in the nucleus, which triggers the transcription of associated target genes [99]. By increasing epithelial-mesenchymal transition (EMT) and collagen deposition, the Wnt/β-catenin signal pathway, which is abnormally active in activated HSCs following liver damage, helps reduce liver fibrosis [100].

The CRBP and β-catenin connection is broken down by the small molecular inhibitor ICG001. In a mouse model of liver fibrosis induced by CCl_4_, ICG001 significantly reduced HSC activation and ECM buildup. It also stopped macrophages from migrating and drastically cut CCL12 production [101]. The CRBP/β-catenin inhibitor PRI-724 attenuated HCV-induced liver fibrosis in mice by inhibiting HSC activation [102]. Octreotide is a somatostatin analog. It inhibited LX2 activation and proliferation, reduced Wnt1 and β-catenin expression *in vitro* and *in vivo*, and lowered CCl_4_-induced liver fibrosis in rats [22].

#### Inhibition of type I collagen synthesis

Up to 50% of the dry weight of the liver in people with liver cirrhosis is made up of collagen [103]. The most prevalent collagen in fibrotic livers is collagen type I. Additionally, the enzyme known as lysyl oxidase-like-2 (LOXL2) alters the cross-linking of type I collagen, leading to an elevation in its levels [104]. Lysyl oxidase (LOX) is a crucial player in collagen stability in chronic liver disease models in animals [105], and chronic human liver disorders have been linked to the overexpression of LOX enzymes [106]. These data gave justification for examining the function of an anti-LOX2 in the management of liver fibrosis. Simtuzumab (SIM) is a humanized antibody that can inhibit collagen cross-linking by targeting LOX2. Unfortunately, data from (phase II) clinical trials on NASH patients with liver F3 (NCT01672866) fibrosis and F4 cirrhosis (NCT01672879) revealed that SIM treatment was not able to reduce hepatic collagen or hepatic venous pressure [107].

Collagen production may be inhibited by knocking down Hsp47, a Col1 chaperone, with small interfering RNA. In three *in-vivo* models of liver fibrosis, Sato et al. found significant anti-fibrotic effects using vitamin A-coupled liposomes carrying Hsp47 siRNA, mostly taken up by HSCs [108]. BMS 986263, a lipid-based nanoparticle designed to deliver Hsp47 siRNA, underwent human trials and demonstrated its safety [109].

### Immune modulation

Fibrosis is caused by inflammatory cells invading the liver, notably macrophages. Kupffer cells, resident macrophages in the liver, can become more activated in response to pathogen-associated molecular patterns (PAMPs) and DAMPs, which can cause inflammatory and immune reactions in the liver. Activated Kupffer cells cause liver inflammation and fibrosis by producing chemokines like CCL2 and CCL5, which bind to their corresponding receptors, C-C chemokine receptors 2 and 5 (CCR2 and CCR5). These chemokines promote HSC activation [110]. Damage to the liver triggers the production of CCL2 from Kupffer cells, which recruits monocytes and promotes their maturation into inflammatory LY6C(high) macrophages [111]. Since macrophages initiate the pro-inflammatory response to liver damage, they are particularly interesting [112]. Therapeutic strategies to reduce fibrosis may 1 day focus on influencing patients’ first innate immune response. In general, anti-inflammatory therapies can be categorized into three main groups. The first group focuses on inhibiting the arrival of inflammatory cells, while the second group aims to reduce macrophage activity. The third group is dedicated to regulating macrophage function and polarization. These approaches are typically given priority when developing treatments for inflammation. It’s important to note that targeting these different aspects of the inflammatory process can provide a multifaceted strategy for managing inflammation-related conditions [10].

#### Preventing recruitment of inflammatory cells

Cenicriviroc (CVC), a dual CCR2/CCR5 inhibitor, decreased liver fibrosis in animal models by reducing the recruitment of pro-inflammatory macrophages [113]. Patients suffering from NASH who took part in the CENTAUR trial “NCT02217475” or the AURORA study of phase III clinical trial that concluded the excellent safety profile of CVC in treating patients suffering from liver fibrosis caused by NASH “NCT03028740” [114]. Furthermore, research is being done on a medication that combines CVC with tropifexor (an FXR agonist), which has been shown to reduce inflammation in NASH animal models. Patients with NASH and liver fibrosis (F2 or F3) participate in a phase 2 study [115].

#### Reducing macrophage activity

The pathogenesis of liver fibrosis involves galectin-3, mainly released by activated macrophages [116]. Belapectin (or GRMD-02), an inhibitor of galectin-3, has been shown to have anti-fibrotic solid effects in mice and rat models of liver fibrosis [117]. Cirrhosis caused by non-alcoholic fatty liver disease is the focus of a current phase 2b/3 clinical investigation “NCT04365868”. In addition, a phase 1 trial looks at the safety and acceptability of GB1211, another galectin-3 receptor inhibitor “NCT03809052” [38].

#### Promotion of macrophage polarization

Promoting a shift from a pathogenic to a restorative phenotype can speed up the recession of fibrosis and encourage liver regeneration [118]. This can be accomplished using pharmacological agents that stimulate macrophage polarization. Macrophage reprogramming in liver illnesses has been studied with many agents, including prostaglandin E2 (PGE2), colony-stimulating factor 1 receptor (CSF-1R), steroids (e.g., dexamethasone), Interleukin 4 (IL-4) and interleukin 10 (IL-10) and secretory leukocyte protease inhibitor (SLPI) agonists [119]. Furthermore, nanotechnology [120] uniquely transforms macrophages into repair cells [119].

### Target receptor-ligand interactions and intracellular signaling

Identifying membrane and nuclear receptors expressed by HSCs has opened up new avenues for anti-fibrotic treatments. Here’s some information about the significance of these receptors and their potential as therapeutic targets: Nuclear Receptors (NRs) are a family of ligand-activated transcription factors that play a role in various biological processes, including the function and development of cells within the hematopoietic and immune systems [121]. They are classified into six subfamilies and comprise a DNA-binding domain (DBD) and a ligand-binding domain (LBD). In the context of liver fibrosis, NRs have been implicated in regulating HSC activation and fibrogenesis [122, 123]. In addition to NRs, membrane Receptors expressed by HSCs have also been identified as potential targets for anti-fibrotic therapies. These receptors, such as G protein-coupled receptors (GPCRs), can activate intracellular signaling pathways and modulate HSC activation and fibrogenesis.

Targeting these receptors with specific agonists or antagonists may help regulate HSC activation and fibrogenesis, leading to the development of novel anti-fibrotic treatments. For example, activation of the farnesoid X receptor (FXR), a nuclear receptor, has been shown to inhibit HSC activation and reduce liver fibrosis in preclinical models. While identifying these receptors has provided valuable insights into the pathogenesis of liver fibrosis, there are still challenges to overcome in developing effective and safe anti-fibrotic therapies. Further research is needed to understand the mechanisms of action of these receptors fully and to explore their potential as therapeutic targets in liver diseases [123].

#### Neurochemical receptors

In HCV patients, regular cannabis usage is a risk factor for increased liver fibrosis [124]. In liver fibrosis, cannabinoid receptors CB1 and CB2 are overexpressed. Compared to CB2 knockout mice (KO) mice, CB1 KO mice show less liver fibrosis [125]. Hepatic stellate cells are turned into myofibroblasts by CB1 agonists. Experimental liver fibrosis is inhibited and reversed by CB1 receptor agonists like Rimonabant [126]. Without causing depression, a peripherally acting CB1 antagonist could treat liver fibrosis.

#### The renin–angiotensin system (RAS)

An important component of fibrogenesis is angiotensin II (Ang II). HSCs release Ang II, which binds to the AT1 receptor that is also expressed by HSCs [127]. Human HSCs are induced to contract and proliferate, and collagen I gene expression is elevated *in vitro* due to Ang ІІ [128]. Inflammation, fibrosis, and lipid peroxidation products after bile duct legation were all reduced in AT1a receptor mutant animals [129]. In light of this, inhibiting the RAS with ACE inhibitors or AT1 receptor blockers may be a successful method for treating liver fibrosis. Losartan treatment for a prolonged period of time reduces nicotinamide adenine dinucleotide phosphate (NADPH) oxidase, inflammation, and fibrogenesis in chronic HCV patients [130].

#### Endothelin 1

The stimulation of HSCs contractility by endothelin may contribute to HSCs activation. An experimental hepatic fibrosis model showed anti-fibrotic action and decreased the level of stellate cell activation by blocking the endothelin receptor with the drug bosentan [131]. Despite initial enthusiasm, bosentan’s development for this application was hindered by signs of hepatotoxicity.

#### Tyrosine kinase receptors

Numerous growth-promoting cytokines interact with cells through tyrosine kinase receptors, including PDGF, fibroblast growth factor (FGF), and transforming growth factor alpha (TGF-α). These receptors are a group of surface molecules that add phosphate groups to specific tyrosine residues upon binding with their respective ligands. HSC proliferation is decreased via the antagonism of pathways that mediate PDGF or vascular endothelial growth factor (VEGF) signals [132]. For instance, multiple receptor tyrosine kinase inhibitors such as sorafenib target the PDGF receptor and have anti-fibrotic effects in animal models [133].

### Gene therapy

SiRNA of 21–23 nucleotides is used in the RNA intervention (RNAi) as a method for precisely silencing individual genes [134]. The direct deletion of TGF-β1 using siRNA has been shown to markedly diminish the expression of α-SMA and collagen type I in HSC-T6 cells and to have anti-fibrotic effects in mice and rats with CCl4-induced hepatic fibrosis [135]. Histone deacetylase 2 (HDAC2) is an up-regulated protein in fibrotic liver tissues caused by CCl4 or HSCs treated with TGF-β1. In HSC-T6 cells treated with TGF-β1, siRNA against HDAC2 expression reduced the expression of collagen type I alpha 1 (COL1a1) and α-SMA [136]. By slowing down the Wnt/β-catenin signal pathway, inhibiting the proliferation of HSC-T6 cells, and inducing apoptosis, β-catenin siRNA slowed the advancement of hepatic fibrosis [99]. That implies that β-catenin siRNA offers a novel method for managing liver fibrosis [137].

In addition to siRNA-based therapy, microRNA (miRNA) may be used to treat liver fibrosis. One form of endogenous non-coding short RNA called miRNA controls the expression of RNA after transcription. In a clinical trial “NCT01200420” of hepatitis C infection, miravirsen (SPC3649), nucleotides, and DNA mixture successfully stopped miR-122 from doing its job and decreased hepatic fibrosis [138]. Mice with hepatic fibrosis caused by CCl4 had markedly better liver function after being treated with the small non-coding RNA MiR-101, which controls the MAPK response. By suppressing the levels of α-SMA and COL1a1, lowering the accumulation of ECM components, and blocking the phosphatidylinositol-3-kinase (PI3K)/Akt/the mammalian target of rapamycin (mTOR) signal pathway, MiR-101 inhibited liver fibrosis and protected liver parenchyma from injury [139].

### Monoclonal antibodies

Monoclonal antibodies are relatively new and developing, becoming crucial to modern pharmacology [140]. Using monoclonal antibodies has fewer adverse side effects and, by itself or in conjunction with other medications, can produce significant results. Many monoclonal antibody treatments with clinical approval are available for a variety of diseases, and they can be used alone or in combination with other therapy (e.g., cetuximab [141], herceptin with docetaxel or paclitaxel [140]. While monoclonal antibodies are a relatively recent strategy for treating liver fibrosis, the field is still in the early stages. Nevertheless, several formulations reached clinical assessment.

### Phytomedicines with multidimensional liver fibrosis effects

Without briefly addressing herbal substances, some of which have shown promise in numerous studies, a review of hepatic anti-fibrotic therapy would not be complete. Several investigations examined Phytodrugs and herbal formulations for treating liver fibrosis. The Phytodrugs with the most significant research into their potential anti-fibrotic effect are resveratrol, silymarin, and curcumin [142, 143].

In animal studies, resveratrol treatment ameliorated steatosis and persistent hepatic disease [144]. Significant protective benefits on indices of liver inflammation and the degree of hepatic steatosis, but not on fibrosis, were seen in NAFLD patients in a randomized, double-blind clinical study comparing oral resveratrol supplementation to a placebo for 12 weeks [145].

A natural herbal flavonoid compound called silymarin (Silybum marianum) is derived from cardoon milk. In a study using cultured human liver HSCs, silybin was discovered to inhibit the pro-fibrogenic actions of HSCs, such as cell proliferation, cell motility, and the production of extracellular matrix components [146]. In non-cirrhotic individuals with NASH, the NAFLD Activity Score showed no statistically marked improvement while being safe, according to a recently finished phase 2 trial titled (SyNCH; NCT00680407) [147].

Curcumin, the main component of *Curcuma longa*, has been studied in many medical conditions and has been found to have anti-inflammatory effects in chronic liver disease antiviral and tumor-preventive properties [148]. Thus, in NASH *in-vivo* models, curcumin treatment suppressed hepatic inflammation, steatosis, formation, and progression of fibrosis [149]. In a rat model of CCl4-induced hepatic fibrosis, curcumin was found to stop HSCs via inducing apoptosis and to prevent liver fibrosis [150]. A complete list of herbal remedies for liver fibrosis is more thoroughly reviewed by Latief and Ahmad (Table 1) [142].

### Combination therapies

Due to the complexity of the etiology, combination therapy that affects two or more targets is likely necessary. The following theories for combination medications have been demonstrated to increase efficacy, and decrease associated adverse effects of single therapies (1): drugs that target several pathways (2); medicines that target diverse characterizations of the disease (3); drugs that combine small-molecule and macromolecular drugs; and (4) drugs that target metabolism and liver fibrosis [151].

Mechanistically complementary, as in the case of participants with NAFLD receiving both an acetyl-CoA carboxylase (ACC1/2) inhibitor (PF-05221304) and diacylglycerol acyltransferase-2 (DGAT2) inhibitor (PF-06865571) [152]. Co-administration of PF-05221304 and PF-06865571 may be a practical strategy. Also, targeting several aspects of a disease, such as FXR (which targets fibrosis and inflammation) and ACC (which decreases fat formation): Patients with NASH showed improvements in hepatic steatosis, biochemistry, and stiffness when receiving a combination of the ACC inhibitor GS-0976 (firsocostat) and the nonsteroidal FXR agonist GS-9674 (cilofexor) (NCT02781584) [153].

Besides, the combination of the small-molecule medications cenicriviroc (CVC) and tropifexor (LJN452) for patients with NASH and liver fibrosis (NCT03517540) offers additional benefits compared to monotherapy [154]. Similarly, small-molecule drug medications combined with macromolecular drugs: In patients with NASH, the FXR agonist cilofexor (GS-9674), ACC inhibitor GS-0976 (firsocostat), and the glucagon-like peptide-1 (GLP-1) receptor agonist semaglutide has shown quiet promising results in countering the progression of the disease evaluated (NCT03987074) [155].

## Discussion

This review delved into the diverse hepatic cell types implicated in liver fibrosis and explored numerous promising treatment avenues. Its overarching goal is to guide further research in this field. Initially, early research focused on the activation of HSCs and collagen deposition as primary points of interest in understanding fibrosis mechanisms. However, recent investigations have broadened their focus to encompass metabolic processes, HSC proliferation, apoptosis, and epigenetic modifications. These studies have significantly expanded our comprehension of fibrosis pathogenesis, opening up exciting new avenues for research into anti-fibrotic therapies.

Despite these advancements, most anti-fibrosis medications, whether designed for liver fibrosis induced by chronic liver disease or other factors, remain in the preclinical development stage. However, there is a glimmer of hope as certain drugs, boasting proven anti-fibrosis efficacy, excellent safety profiles, and patient tolerance, have progressed to the clinical development phase.

Yet, tackling the complexity of fibrosis emergence and progression presents a formidable challenge. Fibrosis is a multifactorial, multistep process, which makes achieving therapeutic progress through targeting a single element, pathway, or link quite challenging. Therefore, the most promising approach lies in developing combination therapies that address multiple pathways simultaneously. This comprehensive strategy should encompass both direct and indirect anti-fibrotic treatments, complemented by therapeutic measures aimed at managing or alleviating the underlying primary disease. In essence, a multifaceted treatment approach offers the best chance of combatting the intricate web of fibrosis.

## Figures and Tables

**Table:** 

**ACE-I**	Angiotensin-converting enzyme inhibitors
**ACC1/2**	Acetyl-CoA carboxylase 1/2
**aHSCs**	Activated HSCs
**ALP**	Alkaline phosphatase
**ALT**	Alanine transaminase
**Ang II**	Angiotensin II
**ASK1**	Apoptosis-signal-regulating kinase 1
**AST**	Aspartate transaminase
**BDL**	Bile duct ligation
**CB1**	Cannabinoid receptors CB1
**CB2**	Cannabinoid receptors CB2
**CCL2**	Chemokine (C-C motif) ligand 2
**CCl4**	Carbon tetrachloride
**CCL5**	Chemokine (C-C motif) ligand 5
**COL1a1**	Collagen type I alpha 1
**COX**	Cycloxygenase 2
**CREB**	Cyclic AMP response element-binding protein
**CSF-1R**	Colony-stimulating factor 1 receptor
**CTGF**	Connective tissue growth factor
**CVC**	Cenicriviroc
**DAMPs**	Damage-linked molecular patterns
**DGAT2**	Diacylglycerol acyltransferase-2
**DMN**	Dimethylnitrosamine
**DPP4**	Dipeptidyl peptidase 4
**ECM**	Extracellular matrix
**EMT**	Epithelial-mesenchymal transition
**FAK**	Focal adhesion kinase
**FasL**	Fas ligand
**FGF**	Fibroblast growth factor
**FN**	Fibronectin
**TIMPs**	Tissue inhibitors of metalloproteinases
**TNF-α**	Tumor necrosis factor-alpha
**FXR**	Farnesoid X Receptor
**Gal-3**	Galectin-3
**GFT505**	Elafibranor
**GLP-1**	glucagon-like peptide-1
**GPx**	Glutathione peroxidase
**GRMD-02**	Belapectin
**GSH**	Glutathione
**HBV**	Hepatitis B virus
**HCC**	Hepatocellular carcinoma
**HCV**	Hepatitis C virus
**HDAC2**	Histone deacetylase 2
**HMG-CoA**	Hydroxymethylglutaryl-coenzyme A
**HSCs**	Hepatic stellate cells
**ICAM-1**	Intercellular adhesion molecule 1
**IL-1**	Interleukin 1
**IL-10**	Interleukin 10
**IL-1β**	Interleukin-1 beta
**IL-4**	Interleukin 4
**IL-6**	Interleukin 6
**iNOS**	Inducible nitric oxide synthase
**KO**	Knockout mice
**LOX**	Lysyl oxidase
**LOXL2**	Lysyl oxidase-like-2
**LRP**	Lipoprotein receptor-related protein −5/6
**MAPK**	Mitogen-activated protein kinase
**MAT 1A**	Methionine adenosyltransferase 1 alpha
**MDA**	Malondialdehyde
**MELD**	Model for End-Stage Liver Disease
**MFA**	Methyl ferulic acid
**miRNA**	MicroRNA
**TRAIL**	Tumor necrosis factor-related apoptosis-inducing ligand
**VEGF**	Vascular endothelial growth factor
**MMP-1**	Matrix metalloproteinase 1
**MMPs**	Matrix metalloproteinases
**NAC**	N-acetylcysteine
**NADPH**	Nicotinamide adenine dinucleotide phosphate
**NAFLD**	Non-alcoholic fatty liver disease
**NASH**	Non-alcoholic steatohepatitis
**NFκB**	Nuclear factor kappa
**NOX**	NADPH oxidase
**OCA**	Obeticholic acid
**PAI-1**	Plasminogen activator inhibitor 1
**PAMPs**	Pathogen-associated molecular patterns
**PDGF**	Platelet-derived growth factor
**PFD**	Pirfenidone
**PGE2**	Prostaglandin E2
**PINP**	Type 1 procollagen peptide
**PPARs**	Peroxisome proliferator-activated receptors
**PPARγ**	Peroxisome proliferator-activated receptor gamma
**qHSCs**	Quiescent HSCs
**RAS**	Renin-angiotensin system
**RBO**	Rice bran oil
**ROS**	Reactive oxygen species
**SAC**	S-allyl cysteine
**SAMe**	S-adenosyl-L-methionine
**SHP**	Small heterodimer partner
**SLPI**	Secretory leukocyte protease inhibitor
**SOD**	Superoxide dismutase
**TAA**	Thioacetamide
**TAZ**	Transcriptional coactivator with PDZ-binding motif
**TGF-α**	Transforming growth factor alpha
**TGF-β1**	Transforming growth factor beta 1
**YAP**	Yes-associated protein
**α-SMA**	α-smooth muscle actin

## References

[1] Higashi T Friedman SL Hoshida Y. Hepatic stellate cells as key target in liver fibrosis. Adv Drug Deliv Rev (2017) 121:27–42. 10.1016/j.addr.2017.05.007 28506744 PMC5682243

[2] Venkatesh SK Torbenson MS. Liver fibrosis quantification. Abdom Radiol (Ny) (2022) 47(3):1032–52. 10.1007/s00261-021-03396-y 35022806 PMC9538706

[3] Tsochatzis EA Bosch J Burroughs AK. Liver cirrhosis. The Lancet (2014) 383(9930):1749–61. 10.1016/s0140-6736(14)60121-5 24480518

[4] Lee YA Wallace MC Friedman SL. Pathobiology of liver fibrosis: a translational success story. Gut (2015) 64(5):830–41. 10.1136/gutjnl-2014-306842 25681399 PMC4477794

[5] Zhang J Liu Q He J Li Y. Novel therapeutic targets in liver fibrosis. Front Mol Biosci (2021) 8:766855. 10.3389/fmolb.2021.766855 34805276 PMC8602792

[6] Asrani SK Devarbhavi H Eaton J Kamath PS. Burden of liver diseases in the world. J Hepatol (2019) 70(1):151–71. 10.1016/j.jhep.2018.09.014 30266282

[7] Boyer-Diaz Z Aristu-Zabalza P Andrés-Rozas M Robert C Ortega-Ribera M Fernández-Iglesias A Pan-PPAR agonist lanifibranor improves portal hypertension and hepatic fibrosis in experimental advanced chronic liver disease. J Hepatol (2021) 74(5):1188–99. 10.1016/j.jhep.2020.11.045 33278455

[8] Iredale J Campana L. Regression of liver fibrosis. Semin Liver Dis (2017) 37(1):001–10. 10.1055/s-0036-1597816 28201843

[9] Friedman SL. Hepatic stellate cells: protean, multifunctional, and enigmatic cells of the liver. Physiol Rev (2008) 88(1):125–72. 10.1152/physrev.00013.2007 18195085 PMC2888531

[10] Wen Y Lambrecht J Ju C Tacke F. Hepatic macrophages in liver homeostasis and diseases-diversity, plasticity and therapeutic opportunities. Cell Mol Immunol (2021) 18(1):45–56. 10.1038/s41423-020-00558-8 33041338 PMC7852525

[11] Aydın MM Akçalı KC. Liver fibrosis. Turk J Gastroenterol (2018) 29(1):14–21. 10.5152/tjg.2018.17330 29391303 PMC6322608

[12] Roehlen N Crouchet E Baumert TF. Liver fibrosis: mechanistic concepts and therapeutic perspectives. Cells (2020) 9(4):875. 10.3390/cells9040875 32260126 PMC7226751

[13] Li MO Wan YY Sanjabi S Robertson AK Flavell RA. Transforming growth factor-beta regulation of immune responses. Annu Rev Immunol (2006) 24:99–146. 10.1146/annurev.immunol.24.021605.090737 16551245

[14] Sun Y Liu B Xie J Jiang X Xiao B Hu X Aspirin attenuates liver fibrosis by suppressing TGF-β1/Smad signaling. Mol Med Rep (2022) 25(5):181. 10.3892/mmr.2022.12697 35322863 PMC8972277

[15] Tan Y Zhen Q Ding X Shen T Liu F Wang Y Association between use of liraglutide and liver fibrosis in patients with type 2 diabetes. Front Endocrinol (Lausanne) (2022) 13:935180. 10.3389/fendo.2022.935180 36034438 PMC9399467

[16] Gu L Zhu Y Lee M Nguyen A Ryujin NT Huang JY Angiotensin II receptor inhibition ameliorates liver fibrosis and enhances hepatocellular carcinoma infiltration by effector T cells. Proc Natl Acad Sci U S A (2023) 120(19):e2300706120. 10.1073/pnas.2300706120 37126700 PMC10175751

[17] Du J Ma YY Yu CH Li YM. Effects of pentoxifylline on nonalcoholic fatty liver disease: a meta-analysis. World J Gastroenterol (2014) 20(2):569–77. 10.3748/wjg.v20.i2.569 24574727 PMC3923033

[18] Salah MM Ashour AA Abdelghany TM Abdel-Aziz AH Salama SA. Pirfenidone alleviates concanavalin A-induced liver fibrosis in mice. Life Sci (2019) 239:116982. 10.1016/j.lfs.2019.116982 31639402

[19] Niu X Hu T Hong Y Li X Shen Y. The role of praziquantel in the prevention and treatment of fibrosis associated with schistosomiasis: a review. J Trop Med (2022) 2022:1413711–8. 10.1155/2022/1413711 36313856 PMC9616668

[20] Yuan S Wei C Liu G Zhang L Li J Li L Sorafenib attenuates liver fibrosis by triggering hepatic stellate cell ferroptosis via HIF-1α/SLC7A11 pathway. Cell Prolif (2022) 55(1):e13158. 10.1111/cpr.13158 34811833 PMC8780895

[21] Musso G Cassader M Paschetta E Gambino R. Pioglitazone for advanced fibrosis in nonalcoholic steatohepatitis: new evidence, new challenges. Hepatology (2017) 65(3):1058–61. 10.1002/hep.28960 27880988

[22] Zhang C Liu XQ Sun HN Meng XM Bao YW Zhang HP Octreotide attenuates hepatic fibrosis and hepatic stellate cells proliferation and activation by inhibiting Wnt/β-catenin signaling pathway, c-Myc and cyclin D1. Int Immunopharmacology (2018) 63:183–90. 10.1016/j.intimp.2018.08.005 30098497

[23] Francis P Forman LM. Statins show promise against progression of liver disease. Clin Liver Dis (2021) 18(6):280–7. 10.1002/cld.1143 PMC8688902 34976372

[24] Doustmohammadian A Nezhadisalami A Safarnezhad Tameshke F Motamed N Maadi M Farahmand M A randomized triple-blind controlled clinical trial evaluation of sitagliptin in the treatment of patients with non-alcoholic fatty liver diseases without diabetes. Front Med (Lausanne) (2022) 9:937554. 10.3389/fmed.2022.937554 35966875 PMC9365981

[25] Wei Z Zhao D Zhang Y Chen Y Zhang S Li Q Rosiglitazone ameliorates bile duct ligation-induced liver fibrosis by down-regulating NF-κB-TNF-α signaling pathway in a PPARγ-dependent manner. Biochem Biophysical Res Commun (2019) 519(4):854–60. 10.1016/j.bbrc.2019.09.084 31561855

[26] Elbaset MA Mohamed BM Gad SA Afifi SM Esatbeyoglu T Abdelrahman SS Erythropoietin mitigated thioacetamide-induced renal injury via JAK2/STAT5 and AMPK pathway. Sci Rep (2023) 13(1):14929. 10.1038/s41598-023-42210-1 37697015 PMC10495371

[27] Elbaset MA Mohamed BM Moustafa PE Mansour DF Afifi SM Esatbeyoglu T Erythropoietin suppresses the hepatic fibrosis caused by thioacetamide: role of the PI3K/akt and TLR4 signaling pathways, Oxid Med Cell Longev, (2023) 2023:5514248. 10.1155/2023/5514248 37649466 PMC10465256

[28] Anstee QM Neuschwander-Tetri BA Wong VW Abdelmalek MF Younossi ZM Yuan J Cenicriviroc for the treatment of liver fibrosis in adults with nonalcoholic steatohepatitis: AURORA Phase 3 study design. Contemp Clin Trials (2020) 89:105922. 10.1016/j.cct.2019.105922 31881392

[29] Karim G Bansal MB. Resmetirom: an orally administered, smallmolecule, liver-directed, beta-selective THR agonist for the treatment of non-alcoholic fatty liver disease and non-alcoholic steatohepatitis. Eur Endocrinol (2023) 19(1):60–70. 10.17925/ee.2023.19.1.60 PMC10258622 37313239

[30] Mu LY Li SQ Tang LX Li R. Efficacy and safety of emricasan in liver cirrhosis and/or fibrosis. Clinics (Sao Paulo). (2021) 76:e2409. 10.6061/clinics/2021/e2409 34133478 PMC8183342

[31] Yoon YC Fang Z Lee JE Park JH Ryu JK Jung KH Selonsertib inhibits liver fibrosis via downregulation of ASK1/MAPK pathway of hepatic stellate cells. Biomolecules Ther (2020) 28(6):527–36. 10.4062/biomolther.2020.016 PMC7585640 32451370

[32] Cai X Liu X Xie W Ma A Tan Y Shang J Hydronidone for the treatment of liver fibrosis related to chronic hepatitis B: a phase 2 randomized controlled trial. Clin Gastroenterol Hepatol (2023) 21(7):1893–901.e7. 10.1016/j.cgh.2022.05.056 35842120

[33] Patel K Harrison SA Elkhashab M Trotter JF Herring R Rojter SE Cilofexor, a nonsteroidal FXR agonist, in patients with noncirrhotic NASH: a phase 2 randomized controlled trial. Hepatology (2020) 72(1):58–71. 10.1002/hep.31205 32115759

[34] Vilar-Gomez E Martinez-Perez Y Calzadilla-Bertot L Torres-Gonzalez A Gra-Oramas B Gonzalez-Fabian L Weight loss through lifestyle modification significantly reduces features of nonalcoholic steatohepatitis. Gastroenterology (2015) 149(2):367–78. quiz e14-5. 10.1053/j.gastro.2015.04.005 25865049

[35] Marcellin P Gane E Buti M Afdhal N Sievert W Jacobson IM Regression of cirrhosis during treatment with tenofovir disoproxil fumarate for chronic hepatitis B: a 5-year open-label follow-up study. The Lancet (2013) 381(9865):468–75. 10.1016/s0140-6736(12)61425-1 23234725

[36] D'Ambrosio R Aghemo A Rumi MG Ronchi G Donato MF Paradis V A morphometric and immunohistochemical study to assess the benefit of a sustained virological response in hepatitis C virus patients with cirrhosis. Hepatology (2012) 56(2):532–43. 10.1002/hep.25606 22271347

[37] Bansal MB Chamroonkul N. Antifibrotics in liver disease: are we getting closer to clinical use? Hepatol Int (2019) 13(1):25–39. 10.1007/s12072-018-9897-3 30302735

[38] Odagiri N Matsubara T Sato-Matsubara M Fujii H Enomoto M Kawada N. Anti-fibrotic treatments for chronic liver diseases: the present and the future. Clin Mol Hepatol (2021) 27(3):413–24. 10.3350/cmh.2020.0187 33317250 PMC8273638

[39] Schwabe RF Luedde T. Apoptosis and necroptosis in the liver: a matter of life and death. Nat Rev Gastroenterol Hepatol (2018) 15(12):738–52. 10.1038/s41575-018-0065-y 30250076 PMC6490680

[40] Thapaliya S Wree A Povero D Inzaugarat ME Berk M Dixon L Caspase 3 inactivation protects against hepatic cell death and ameliorates fibrogenesis in a diet-induced NASH model. Dig Dis Sci (2014) 59(6):1197–206. 10.1007/s10620-014-3167-6 24795036 PMC4512760

[41] Gracia-Sancho J Manicardi N Ortega-Ribera M Maeso-Díaz R Guixé-Muntet S Fernández-Iglesias A Emricasan ameliorates portal hypertension and liver fibrosis in cirrhotic rats through a hepatocyte-mediated paracrine mechanism. Hepatol Commun (2019) 3(7):987–1000. 10.1002/hep4.1360 31304452 PMC6601324

[42] Harrison SA Goodman Z Jabbar A Vemulapalli R Younes ZH Freilich B A randomized, placebo-controlled trial of emricasan in patients with NASH and F1-F3 fibrosis. J Hepatol (2020) 72(5):816–27. 10.1016/j.jhep.2019.11.024 31887369

[43] Barreyro FJ Holod S Finocchietto PV Camino AM Aquino JB Avagnina A The pan-caspase inhibitor emricasan (IDN-6556) decreases liver injury and fibrosis in a murine model of non-alcoholic steatohepatitis. Liver Int (2015) 35(3):953–66. 10.1111/liv.12570 24750664

[44] Eguchi A Koyama Y Wree A Johnson CD Nakamura R Povero D Emricasan, a pan-caspase inhibitor, improves survival and portal hypertension in a murine model of common bile-duct ligation. J Mol Med (2018) 96:575–83. 10.1007/s00109-018-1642-9 29728708 PMC6347405

[45] Garcia-Tsao G Fuchs M Shiffman M Borg BB Pyrsopoulos N Shetty K Emricasan (IDN-6556) lowers portal pressure in patients with compensated cirrhosis and severe portal hypertension. Hepatology (2019) 69(2):717–28. 10.1002/hep.30199 30063802 PMC6587783

[46] Manka P Zeller A Syn W-K. Fibrosis in chronic liver disease: an update on diagnostic and treatment modalities. Drugs (2019) 79(9):903–27. 10.1007/s40265-019-01126-9 31119644

[47] Matsukawa J Matsuzawa A Takeda K Ichijo H. The ASK1-MAP kinase cascades in mammalian stress response. J Biochem (2004) 136(3):261–5. 10.1093/jb/mvh134 15598880

[48] Budas G Karnik S Jonnson T Shafizadeh T Watkins S Breckenridge D Reduction of liver steatosis and fibrosis with an Ask1 inhibitor in a murine model of nash is accompanied by improvements in cholesterol, bile acid and lipid metabolism. J Hepatol (2016) 64(64):S170. 10.1016/s0168-8278(16)01686-x

[49] Loomba R Lawitz E Mantry PS Jayakumar S Caldwell SH Arnold H The ASK1 inhibitor selonsertib in patients with nonalcoholic steatohepatitis: a randomized, phase 2 trial. Hepatology (2018) 67(2):549–59. 10.1002/hep.29514 28892558 PMC5814892

[50] Luedde T Assmus U Wüstefeld T Meyer zu Vilsendorf A Roskams T Schmidt-Supprian M Deletion of IKK2 in hepatocytes does not sensitize these cells to TNF-induced apoptosis but protects from ischemia/reperfusion injury. J Clin Invest (2005) 115(4):849–59. 10.1172/jci23493 15776110 PMC1064982

[51] Yang YM Seki E. TNFα in liver fibrosis. Curr Pathobiol Rep (2015) 3(4):253–61. 10.1007/s40139-015-0093-z 26726307 PMC4693602

[52] Flores-Contreras L Sandoval-Rodríguez AS Mena-Enriquez MG Lucano-Landeros S Arellano-Olivera I Alvarez-Álvarez A Treatment with pirfenidone for two years decreases fibrosis, cytokine levels and enhances CB2 gene expression in patients with chronic hepatitis C. BMC Gastroenterol (2014) 14:131. 10.1186/1471-230x-14-131 25064094 PMC4236537

[53] Komiya C Tanaka M Tsuchiya K Shimazu N Mori K Furuke S Antifibrotic effect of pirfenidone in a mouse model of human nonalcoholic steatohepatitis. Sci Rep (2017) 7:44754. 10.1038/srep44754 28303974 PMC5355985

[54] Poo JL Torre A Aguilar-Ramírez JR Cruz M Mejía-Cuán L Cerda E Benefits of prolonged-release pirfenidone plus standard of care treatment in patients with advanced liver fibrosis: PROMETEO study. Hepatol Int (2020) 14(5):817–27. 10.1007/s12072-020-10069-3 32813194 PMC7561536

[55] Kodai S Takemura S Kubo S Azuma H Minamiyama Y. Therapeutic administration of an ingredient of aged-garlic extracts, <i&gt;S&lt;/i&gt;-allyl cysteine resolves liver fibrosis established by carbon tetrachloride in rats. J Clin Biochem Nutr (2015) 56(3):179–85. 10.3164/jcbn.14-108 26060347 PMC4454081

[56] Sanyal AJ. ACP journal club: vitamin E, but not pioglitazone, improved nonalcoholic steatohepatitis in nondiabetic patients. Ann Intern Med (2010) 153(6):Jc3–12. 10.7326/0003-4819-153-6-201009210-02012 20855789

[57] Bashandy SAE Ebaid H Al-Tamimi J Hassan I Omara EA Elbaset MA Protective effect of daidzein against diethylnitrosamine/carbon tetrachloride-induced hepatocellular carcinoma in male rats. Biology (2023) 12(9):1184. 10.3390/biology12091184 37759583 PMC10525464

[58] Abdel-Rahman RF Fayed HM Mohamed MA Hessin AF Asaad GF AbdelRahman SS Apigenin role against thioacetamide-triggered liver fibrosis: deciphering the PPARγ/TGF-β1/NF-κB and the HIF/FAK/AKT pathways. J Herbmed Pharmacol (2023) 12:202–13. 10.34172/jhp.2023.21

[59] Ogaly HA Abdel-Rahman RF Mohamed MAE Oa AF Khattab MS Abd-Elsalam RM. Thymol ameliorated neurotoxicity and cognitive deterioration in a thioacetamide-induced hepatic encephalopathy rat model; involvement of the BDNF/CREB signaling pathway. Food Funct (2022) 13:6180–94. 10.1039/d1fo04292k 35583008

[60] Elbaset MA Nasr M Ibrahim BMM Ahmed-Farid OAH Bakeer RM Hassan NS Curcumin nanoemulsion counteracts hepatic and cardiac complications associated with high-fat/high-fructose diet in rats. J Food Biochem (2022) 46:e14442. 10.1111/jfbc.14442 36165438

[61] Khalifa MMAMM Baset MA El-Eraky WI Abdelbaset MMA Safar MM Mahmoud SHSS Promising synthesized bis (arylmethylidene) acetone -polymeric PCL emulsified nanoparticles with enhanced antimicrobial/antioxidant efficacy: *in-vitro* and *in-vivo* evaluation. Egypt J Chem (2022) 11:64–72.

[62] Abdelbaset M Safar MM Mahmoud SS Negm SA Agha AM. Red yeast rice and coenzyme Q10 as safe alternatives to surmount atorvastatin-induced myopathy in hyperlipidemic rats. Can J Physiol Pharmacol (2014) 92:481–9. 10.1139/cjpp-2013-0430 24896301

[63] Abdel-Rahman RF Fayed HM Asaad GF Ogaly HA Hessin AF Salama AAA The involvement of TGF-β1/FAK/α-SMA pathway in the antifibrotic impact of rice bran oil on thioacetamide-induced liver fibrosis in rats. PLoS ONE (2021) 16:e0260130. 10.1371/journal.pone.0260130 34965258 PMC8716044

[64] Abdelmottaleb SAM Ibrahim FAA Elbaset MA Aziz SW Morsy FA Abdellatif N Goldenberry (Physalis peruviana) alleviates hepatic oxidative stress and metabolic syndrome in obese rats. J Appl Pharm Sci (2022) 12. 10.7324/japs.2022.121115

[65] Moussa SAA Ibrahim FAA Elbaset MA Aziz SW Abdellatif NA Attia AM Efficacy of goldenberry extract in chelated iron overload induced by obesity: novel safety concept for the treatment of iron overloads diseases. J Appl Biol Biotechnol (2022) 10:92–100. 10.7324/jabb.2022.100413

[66] Ayoub IM El-Baset MA Elghonemy MM Bashandy SAE Ibrahim FAA Ahmed-Farid OAH Chemical profile of cyperus laevigatus and its protective effects against thioacetamide-induced hepatorenal toxicity in rats. Molecules (2022) 27:6470. 10.3390/molecules27196470 36235007 PMC9573427

[67] Altenhöfer S Kleikers PW Radermacher KA Scheurer P Rob Hermans JJ Schiffers P The NOX toolbox: validating the role of NADPH oxidases in physiology and disease. Cell Mol Life Sci (2012) 69(14):2327–43. 10.1007/s00018-012-1010-9 22648375 PMC3383958

[68] Jiang JX Chen X Serizawa N Szyndralewiez C Page P Schröder K Liver fibrosis and hepatocyte apoptosis are attenuated by GKT137831, a novel NOX4/NOX1 inhibitor *in vivo* . Free Radic Biol Med (2012) 53(2):289–96. 10.1016/j.freeradbiomed.2012.05.007 22618020 PMC3392471

[69] Aoyama T Paik YH Watanabe S Laleu B Gaggini F Fioraso-Cartier L Nicotinamide adenine dinucleotide phosphate oxidase in experimental liver fibrosis: GKT137831 as a novel potential therapeutic agent. Hepatology (2012) 56(6):2316–27. 10.1002/hep.25938 22806357 PMC3493679

[70] Invernizzi P Carbone M Jones D Levy C Little N Wiesel P Setanaxib, a first-in-class selective NADPH oxidase 1/4 inhibitor for primary biliary cholangitis: a randomized, placebo-controlled, phase 2 trial. Liver Int (2023) 43(7):1507–22. 10.1111/liv.15596 37183520

[71] Lee NY Suk KT. The role of the gut microbiome in liver cirrhosis treatment. Int J Mol Sci (2020) 22(1):199. 10.3390/ijms22010199 33379148 PMC7796381

[72] Milosevic I Vujovic A Barac A Djelic M Korac M Radovanovic Spurnic A Gut-liver Axis, gut microbiota, and its modulation in the management of liver diseases: a review of the literature. Int J Mol Sci (2019) 20(2):395. 10.3390/ijms20020395 30658519 PMC6358912

[73] Ahmed LA Salem MB Seif El-Din SH El-Lakkany NM Ahmed HO Nasr SM Gut microbiota modulation as a promising therapy with metformin in rats with non-alcoholic steatohepatitis: role of LPS/TLR4 and autophagy pathways. Eur J Pharmacol (2020) 887:173461. 10.1016/j.ejphar.2020.173461 32758573

[74] Ma YY Li L Yu CH Shen Z Chen LH Li YM. Effects of probiotics on nonalcoholic fatty liver disease: a meta-analysis. World J Gastroenterol (2013) 19(40):6911–8. 10.3748/wjg.v19.i40.6911 24187469 PMC3812493

[75] Chou R Dana T Blazina I Daeges M Jeanne TL. Statins for prevention of cardiovascular disease in adults: evidence report and systematic review for the US preventive services task force. Jama (2016) 316(19):2008–24. 10.1001/jama.2015.15629 27838722

[76] Pose E Trebicka J Mookerjee RP Angeli P Ginès P. Statins: old drugs as new therapy for liver diseases? J Hepatol (2019) 70(1):194–202. 10.1016/j.jhep.2018.07.019 30075229

[77] Dongiovanni P Petta S Mannisto V Mancina RM Pipitone R Karja V Statin use and non-alcoholic steatohepatitis in at risk individuals. J Hepatol (2015) 63(3):705–12. 10.1016/j.jhep.2015.05.006 25980762

[78] Nascimbeni F Aron-Wisnewsky J Pais R Tordjman J Poitou C Charlotte F Statins, antidiabetic medications and liver histology in patients with diabetes with non-alcoholic fatty liver disease. BMJ Open Gastroenterol (2016) 3(1):e000075. 10.1136/bmjgast-2015-000075 PMC4838660 27110380

[79] Cioboată R Găman A Traşcă D Ungureanu A Docea AO Tomescu P Pharmacological management of non-alcoholic fatty liver disease: atorvastatin versus pentoxifylline. Exp Ther Med (2017) 13(5):2375–81. 10.3892/etm.2017.4256 28565851 PMC5443168

[80] Hussien YA Mansour DF Nada SA Abd El-Rahman SS Abdelsalam RM Attia AS Linagliptin attenuates thioacetamide-induced hepatic encephalopathy in rats: modulation of C/EBP-beta and CX3CL1/Fractalkine, neuro-inflammation, oxidative stress and behavioral defects. Life Sci (2022) 295:120378. 10.1016/j.lfs.2022.120378 35134437

[81] Derynck R Zhang YE. Smad-dependent and smad-independent pathways in TGF-beta family signalling. Nature (2003) 425(6958):577–84. 10.1038/nature02006 14534577

[82] Lv X Yao T He R He Y Li M Han Y Protective effect of fluorofenidone against acute lung injury through suppressing the MAPK/NF-κB pathway. Front Pharmacol (2021) 12:772031. 10.3389/fphar.2021.772031 34987397 PMC8721041

[83] Peng Y Li L Zhang X Xie M Yang C Tu S Fluorofenidone affects hepatic stellate cell activation in hepatic fibrosis by targeting the TGF-β1/Smad and MAPK signaling pathways. Exp Ther Med (2019) 18(1):41–8. 10.3892/etm.2019.7548 31258636 PMC6566051

[84] Liu J Kong D Qiu J Xie Y Lu Z Zhou C Praziquantel ameliorates CCl(4) -induced liver fibrosis in mice by inhibiting TGF-β/Smad signalling via up-regulating Smad7 in hepatic stellate cells. Br J Pharmacol (2019) 176(24):4666–80. 10.1111/bph.14831 31412137 PMC6965681

[85] Yang L Chan CC Kwon OS Liu S McGhee J Stimpson SA Regulation of peroxisome proliferator-activated receptor-gamma in liver fibrosis. Am J Physiology-Gastrointestinal Liver Physiol (2006) 291(5):G902–11. 10.1152/ajpgi.00124.2006 16798724

[86] Sanyal AJ Chalasani N Kowdley KV McCullough A Diehl AM Bass NM Pioglitazone, vitamin E, or placebo for nonalcoholic steatohepatitis. N Engl J Med (2010) 362(18):1675–85. 10.1056/nejmoa0907929 20427778 PMC2928471

[87] Musso G Cassader M Paschetta E Gambino R. Thiazolidinediones and advanced liver fibrosis in nonalcoholic steatohepatitis: a meta-analysis. JAMA Intern Med (2017) 177(5):633–40. 10.1001/jamainternmed.2016.9607 28241279 PMC5470366

[88] Cheng HS Tan WR Low ZS Marvalim C Lee JYH Tan NS. Exploration and development of PPAR modulators in health and disease: an update of clinical evidence. Int J Mol Sci (2019) 20(20):5055. 10.3390/ijms20205055 31614690 PMC6834327

[89] Alukal JJ Thuluvath PJ. Reversal of NASH fibrosis with pharmacotherapy. Hepatol Int (2019) 13(5):534–45. 10.1007/s12072-019-09970-3 31363910

[90] Roth JD Veidal SS Fensholdt LKD Rigbolt KTG Papazyan R Nielsen JC Combined obeticholic acid and elafibranor treatment promotes additive liver histological improvements in a diet-induced ob/ob mouse model of biopsy-confirmed NASH. Scientific Rep (2019) 9(1):9046. 10.1038/s41598-019-45178-z PMC6588626 31227742

[91] Schuppan D Ashfaq-Khan M Yang AT Kim YO. Liver fibrosis: direct antifibrotic agents and targeted therapies. Matrix Biol (2018) 68-69:435–51. 10.1016/j.matbio.2018.04.006 29656147

[92] Boeckmans J Natale A Rombaut M Buyl K Rogiers V De Kock J Anti-NASH drug development hitches a lift on PPAR agonism. Cells (2019) 9(1):37. 10.3390/cells9010037 31877771 PMC7016963

[93] Teodoro JS Rolo AP Palmeira CM. Hepatic FXR: key regulator of whole-body energy metabolism. Trends Endocrinol Metab (2011) 22(11):458–66. 10.1016/j.tem.2011.07.002 21862343

[94] Mann J Mann DA. Transcriptional regulation of hepatic stellate cells. Adv Drug Deliv Rev (2009) 61(7-8):497–512. 10.1016/j.addr.2009.03.011 19393271

[95] Zhang S Wang J Liu Q Harnish DC. Farnesoid X receptor agonist WAY-362450 attenuates liver inflammation and fibrosis in murine model of non-alcoholic steatohepatitis. J Hepatol (2009) 51(2):380–8. 10.1016/j.jhep.2009.03.025 19501927

[96] Fiorucci S Rizzo G Antonelli E Renga B Mencarelli A Riccardi L A farnesoid x receptor-small heterodimer partner regulatory cascade modulates tissue metalloproteinase inhibitor-1 and matrix metalloprotease expression in hepatic stellate cells and promotes resolution of liver fibrosis. J Pharmacol Exp Ther (2005) 314(2):584–95. 10.1124/jpet.105.084905 15860571

[97] Verbeke L Farre R Trebicka J Komuta M Roskams T Klein S Obeticholic acid, a farnesoid X receptor agonist, improves portal hypertension by two distinct pathways in cirrhotic rats. Hepatology (2014) 59(6):2286–98. 10.1002/hep.26939 24259407

[98] Mudaliar S Henry RR Sanyal AJ Morrow L Marschall HU Kipnes M Efficacy and safety of the farnesoid X receptor agonist obeticholic acid in patients with type 2 diabetes and nonalcoholic fatty liver disease. Gastroenterology (2013) 145(3):574–82.e1. 10.1053/j.gastro.2013.05.042 23727264

[99] Ge WS Wang YJ Wu JX Fan JG Chen YW Zhu L. β-catenin is overexpressed in hepatic fibrosis and blockage of Wnt/β-catenin signaling inhibits hepatic stellate cell activation. Mol Med Rep (2014) 9(6):2145–51. 10.3892/mmr.2014.2099 24691643 PMC4055486

[100] Nishikawa K Osawa Y Kimura K. Wnt/β-Catenin signaling as a potential target for the treatment of liver cirrhosis using antifibrotic drugs. Int J Mol Sci (2018) 19(10):3103. 10.3390/ijms19103103 30308992 PMC6213128

[101] Akcora B Storm G Bansal R. Inhibition of canonical WNT signaling pathway by β-catenin/CBP inhibitor ICG-001 ameliorates liver fibrosis *in vivo* through suppression of stromal CXCL12. Biochim Biophys Acta (Bba) - Mol Basis Dis (2018) 1864(3):804–18. 10.1016/j.bbadis.2017.12.001 29217140

[102] Tokunaga Y Osawa Y Ohtsuki T Hayashi Y Yamaji K Yamane D Selective inhibitor of Wnt/β-catenin/CBP signaling ameliorates hepatitis C virus-induced liver fibrosis in mouse model. Sci Rep (2017) 7(1):325. 10.1038/s41598-017-00282-w 28336942 PMC5427997

[103] Schuppan D. Structure of the extracellular matrix in normal and fibrotic liver: collagens and glycoproteins. Semin Liver Dis (1990) 10(1):1–10. 10.1055/s-2008-1040452 2186485

[104] Koyama Y Xu J Liu X Brenner DA. New developments on the treatment of liver fibrosis. Dig Dis (2016) 34(5):589–96. 10.1159/000445269 27332862 PMC4961096

[105] Liu SB Ikenaga N Peng Z-W Sverdlov DY Greenstein A Smith V Lysyl oxidase activity contributes to collagen stabilization during liver fibrosis progression and limits spontaneous fibrosis reversal in mice. FASEB J (2016) 30(4):1599–609. 10.1096/fj.14-268425 26700732

[106] Barry-Hamilton V Spangler R Marshall D McCauley S Rodriguez HM Oyasu M Allosteric inhibition of lysyl oxidase-like-2 impedes the development of a pathologic microenvironment. Nat Med (2010) 16(9):1009–17. 10.1038/nm.2208 20818376

[107] Muir AJ Levy C Janssen HLA Montano-Loza AJ Shiffman ML Caldwell S Simtuzumab for primary sclerosing cholangitis: phase 2 study results with insights on the natural history of the disease. Hepatology (2019) 69(2):684–98. 10.1002/hep.30237 30153359

[108] Sato Y Murase K Kato J Kobune M Sato T Kawano Y Resolution of liver cirrhosis using vitamin A-coupled liposomes to deliver siRNA against a collagen-specific chaperone. Nat Biotechnol (2008) 26(4):431–42. 10.1038/nbt1396 18376398

[109] Soule B Tirucherai G Kavita U Kundu S Christian R. Safety, tolerability, and pharmacokinetics of BMS-986263/ND-L02-s0201, a novel targeted lipid nanoparticle delivering HSP47 siRNA, in healthy participants: a randomised, placebo-controlled, double-blind, phase 1 study. J Hepatol (2018) 68:S112. 10.1016/s0168-8278(18)30442-2

[110] Berres ML Koenen RR Rueland A Zaldivar MM Heinrichs D Sahin H Antagonism of the chemokine Ccl5 ameliorates experimental liver fibrosis in mice. J Clin Invest (2010) 120(11):4129–40. 10.1172/jci41732 20978355 PMC2964968

[111] Miura K Yang L van Rooijen N Ohnishi H Seki E. Hepatic recruitment of macrophages promotes nonalcoholic steatohepatitis through CCR2. Am J Physiology-Gastrointestinal Liver Physiol (2012) 302(11):G1310–21. 10.1152/ajpgi.00365.2011 PMC3378163 22442158

[112] Tacke F Zimmermann HW. Macrophage heterogeneity in liver injury and fibrosis. J Hepatol (2014) 60(5):1090–6. 10.1016/j.jhep.2013.12.025 24412603

[113] Lefebvre E Moyle G Reshef R Richman LP Thompson M Hong F Antifibrotic effects of the dual CCR2/CCR5 antagonist cenicriviroc in animal models of liver and kidney fibrosis. PLoS One (2016) 11(6):e0158156. 10.1371/journal.pone.0158156 27347680 PMC4922569

[114] Anstee QM Neuschwander-Tetri BA Wai-Sun Wong V Abdelmalek MF Rodriguez-Araujo G Landgren H Cenicriviroc lacked efficacy to treat liver fibrosis in nonalcoholic steatohepatitis: AURORA phase III randomized study. Clin Gastroenterol Hepatol official Clin Pract J Am Gastroenterological Assoc (2023) 1542-3565(23):00273–2. 10.1016/j.cgh.2023.04.003 37061109

[115] Sanyal AJ Lopez P Lawitz EJ Lucas KJ Loeffler J Kim W Tropifexor for nonalcoholic steatohepatitis: an adaptive, randomized, placebo-controlled phase 2a/b trial. Nat Med (2023) 29(2):392–400. 10.1038/s41591-022-02200-8 36797481 PMC9941046

[116] Sano H Hsu DK Yu L Apgar JR Kuwabara I Yamanaka T Human galectin-3 is a novel chemoattractant for monocytes and macrophages. J Immunol (2000) 165(4):2156–64. 10.4049/jimmunol.165.4.2156 10925302

[117] Traber PG Zomer E. Therapy of experimental NASH and fibrosis with galectin inhibitors. PLoS One (2013) 8(12):e83481. 10.1371/journal.pone.0083481 24367597 PMC3867460

[118] Triantafyllou E Woollard KJ McPhail MJW Antoniades CG Possamai LA. The role of monocytes and macrophages in acute and acute-on-chronic liver failure. Front Immunol (2018) 9:2948. 10.3389/fimmu.2018.02948 30619308 PMC6302023

[119] van der Heide D Weiskirchen R Bansal R. Therapeutic targeting of hepatic macrophages for the treatment of liver diseases. Front Immunol (2019) 10:2852. 10.3389/fimmu.2019.02852 31849997 PMC6901832

[120] Colino CI Lanao JM Gutierrez-Millan C. Targeting of hepatic macrophages by therapeutic nanoparticles. Front Immunol (2020) 11:218. 10.3389/fimmu.2020.00218 32194546 PMC7065596

[121] Chute JP Ross JR McDonnell DP. Minireview: nuclear receptors, hematopoiesis, and stem cells. Mol Endocrinol (2010) 24(1):1–10. 10.1210/me.2009-0332 19934345 PMC2802897

[122] Pastori V Pozzi S Labedz A Ahmed S Ronchi AE. Role of nuclear receptors in controlling erythropoiesis. Int J Mol Sci (2022) 23(5):2800. 10.3390/ijms23052800 35269942 PMC8911257

[123] Wagner M Zollner G Trauner M. Nuclear receptors in liver disease. Hepatology (2011) 53(3):1023–34. 10.1002/hep.24148 21319202

[124] Patsenker E Sachse P Chicca A Gachet MS Schneider V Mattsson J Elevated levels of endocannabinoids in chronic hepatitis C may modulate cellular immune response and hepatic stellate cell activation. Int J Mol Sci (2015) 16(4):7057–76. 10.3390/ijms16047057 25826533 PMC4425004

[125] Pacher P Gao B. Endocannabinoids and liver disease. III. Endocannabinoid effects on immune cells: implications for inflammatory liver diseases. Am J Physiology-Gastrointestinal Liver Physiol (2008) 294(4):G850–4. 10.1152/ajpgi.00523.2007 PMC2376822 18239059

[126] Dai E Zhang J Zhang D Yang L Wang Y Jiang X Rimonabant inhibits proliferation, collagen secretion and induces apoptosis in hepatic stellate cells. Hepatogastroenterology (2014) 61(135):2052–61. 25713910

[127] Bataller R North KE Brenner DA. Genetic polymorphisms and the progression of liver fibrosis: a critical appraisal. Hepatology (2003) 37(3):493–503. 10.1053/jhep.2003.50127 12601343

[128] Bataller R Ginès P Nicolás JM Görbig MN Garcia-Ramallo E Gasull X Angiotensin II induces contraction and proliferation of human hepatic stellate cells. Gastroenterology (2000) 118(6):1149–56. 10.1016/s0016-5085(00)70368-4 10833490

[129] Yang L Bataller R Dulyx J Coffman TM Ginès P Rippe RA Attenuated hepatic inflammation and fibrosis in angiotensin type 1a receptor deficient mice. J Hepatol (2005) 43(2):317–23. 10.1016/j.jhep.2005.02.034 15964094

[130] Ghany MG Kleiner DE Alter H Doo E Khokar F Promrat K Progression of fibrosis in chronic hepatitis C. Gastroenterology (2003) 124(1):97–104. 10.1053/gast.2003.50018 12512034

[131] Rockey DC Chung JJ. Endothelin antagonism in experimental hepatic fibrosis. Implications for endothelin in the pathogenesis of wound healing. J Clin Invest (1996) 98(6):1381–8. 10.1172/jci118925 8823303 PMC507564

[132] Gonzalo T Beljaars L van de Bovenkamp M Temming K van Loenen AM Reker-Smit C Local inhibition of liver fibrosis by specific delivery of a platelet-derived growth factor kinase inhibitor to hepatic stellate cells. J Pharmacol Exp Ther (2007) 321(3):856–65. 10.1124/jpet.106.114496 17369283

[133] Hong F Chou H Fiel MI Friedman SL. Antifibrotic activity of sorafenib in experimental hepatic fibrosis: refinement of inhibitory targets, dosing, and window of efficacy *in vivo* . Dig Dis Sci (2013) 58(1):257–64. 10.1007/s10620-012-2325-y 22918681 PMC3543488

[134] Buchman TG. RNAi. Crit Care Med (2005) 33(12):S441–3. 10.1097/01.ccm.0000191263.35901.5c 16340416

[135] Cheng K Yang N Mahato RI. TGF-β1 gene silencing for treating liver fibrosis. Mol Pharmaceutics (2009) 6(3):772–9. 10.1021/mp9000469 PMC2743970 19388665

[136] Li X Wu XQ Xu T Li XF Yang Y Li WX Role of histone deacetylases(HDACs) in progression and reversal of liver fibrosis. Toxicol Appl Pharmacol (2016) 306:58–68. 10.1016/j.taap.2016.07.003 27396813

[137] Chen SW Wu BY Xu SP Fan KX Yan L Gong Y Suppression of CB1 cannabinoid receptor by lentivirus mediated small interfering RNA ameliorates hepatic fibrosis in rats. PLoS One (2012) 7(12):e50850. 10.1371/journal.pone.0050850 23251393 PMC3520929

[138] Zeng C Wang YL Xie C Sang Y Li TJ Zhang M Identification of a novel TGF-β-miR-122-fibronectin 1/serum response factor signaling cascade and its implication in hepatic fibrogenesis. Oncotarget (2015) 6(14):12224–33. 10.18632/oncotarget.3652 25909171 PMC4494934

[139] Lei Y Wang QL Shen L Tao YY Liu CH. MicroRNA-101 suppresses liver fibrosis by downregulating PI3K/Akt/mTOR signaling pathway. Clin Res Hepatol Gastroenterol (2019) 43(5):575–84. 10.1016/j.clinre.2019.02.003 30857885

[140] Powroźnik B Kubowicz P Pękala E. Monoclonal antibodies in targeted therapy. Postepy Hig Med Dosw (Online) (2012) 66:663–73. 10.5604/17322693.1009980 23001208

[141] Cunningham D Humblet Y Siena S Khayat D Bleiberg H Santoro A Cetuximab monotherapy and cetuximab plus irinotecan in irinotecan-refractory metastatic colorectal cancer. N Engl J Med (2004) 351(4):337–45. 10.1056/nejmoa033025 15269313

[142] Latief U Ahmad R. Herbal remedies for liver fibrosis: a review on the mode of action of fifty herbs. J Traditional Complement Med (2018) 8(3):352–60. 10.1016/j.jtcme.2017.07.002 PMC6035307 29992106

[143] Duval F Moreno-Cuevas JE González-Garza MT Maldonado-Bernal C Cruz-Vega DE. Liver fibrosis and mechanisms of the protective action of medicinal plants targeting inflammation and the immune response. Int J Inflamm (2015) 2015:1–14. 10.1155/2015/943497 PMC4411506 25954568

[144] Kessoku T Imajo K Honda Y Kato T Ogawa Y Tomeno W Resveratrol ameliorates fibrosis and inflammation in a mouse model of nonalcoholic steatohepatitis. Sci Rep (2016) 6:22251. 10.1038/srep22251 26911834 PMC4766502

[145] Faghihzadeh F Adibi P Rafiei R Hekmatdoost A. Resveratrol supplementation improves inflammatory biomarkers in patients with nonalcoholic fatty liver disease. Nutr Res (2014) 34(10):837–43. 10.1016/j.nutres.2014.09.005 25311610

[146] Trappoliere M Caligiuri A Schmid M Bertolani C Failli P Vizzutti F Silybin, a component of sylimarin, exerts anti-inflammatory and anti-fibrogenic effects on human hepatic stellate cells. J Hepatol (2009) 50(6):1102–11. 10.1016/j.jhep.2009.02.023 19398228

[147] Navarro VJ Belle SH D'Amato M Adfhal N Brunt EM Fried MW Silymarin in non-cirrhotics with non-alcoholic steatohepatitis: a randomized, double-blind, placebo controlled trial. PLoS One (2019) 14(9):e0221683. 10.1371/journal.pone.0221683 31536511 PMC6752871

[148] Tu CT Han B Liu HC Zhang SC. Curcumin protects mice against concanavalin A-induced hepatitis by inhibiting intrahepatic intercellular adhesion molecule-1 (ICAM-1) and CXCL10 expression. Mol Cel Biochem (2011) 358(1-2):53–60. 10.1007/s11010-011-0920-4 21695461

[149] Li B Wang L Lu Q Da W. Liver injury attenuation by curcumin in a rat NASH model: an Nrf2 activation-mediated effect? Ir J Med Sci (2016) 185(1):93–100. 10.1007/s11845-014-1226-9 25385666

[150] Shu JC He YJ Lv X Ye GR Wang LX. Curcumin prevents liver fibrosis by inducing apoptosis and suppressing activation of hepatic stellate cells. J Nat Med (2009) 63(4):415–20. 10.1007/s11418-009-0347-3 19554395

[151] Zhang D Zhang Y Sun B. The molecular mechanisms of liver fibrosis and its potential therapy in application. Int J Mol Sci (2022) 23(20):12572. 10.3390/ijms232012572 36293428 PMC9604031

[152] Loomba R Noureddin M Kowdley KV Kohli A Sheikh A Neff G Combination therapies including cilofexor and firsocostat for bridging fibrosis and cirrhosis attributable to NASH. Hepatology (2021) 73(2):625–43. 10.1002/hep.31622 33169409

[153] Pedrosa M Seyedkazemi S Francque S Sanyal A Rinella M Charlton M A randomized, double-blind, multicenter, phase 2b study to evaluate the safety and efficacy of a combination of tropifexor and cenicriviroc in patients with nonalcoholic steatohepatitis and liver fibrosis: study design of the TANDEM trial. Contemp Clin trials (2020) 88:105889. 10.1016/j.cct.2019.105889 31731005

[154] Alkhouri N Herring R Kabler H Kayali Z Hassanein T Kohli A Safety and efficacy of combination therapy with semaglutide, cilofexor and firsocostat in patients with non-alcoholic steatohepatitis: a randomised, open-label phase II trial. J Hepatol (2022) 77(3):607–18. 10.1016/j.jhep.2022.04.003 35439567

[155] Frias JP Nauck MA Van J Kutner ME Cui X Benson C Efficacy and safety of LY3298176, a novel dual GIP and GLP-1 receptor agonist, in patients with type 2 diabetes: a randomised, placebo-controlled and active comparator-controlled phase 2 trial. The Lancet (2018) 392(10160):2180–93. 10.1016/s0140-6736(18)32260-8 30293770

